# Assessment of localisation to auditory stimulation in post-comatose states: use the patient’s own name

**DOI:** 10.1186/1471-2377-13-27

**Published:** 2013-03-18

**Authors:** Lijuan Cheng, Olivia Gosseries, Limei Ying, Xiaohua Hu, Dan Yu, Hongxing Gao, Minhui He, Caroline Schnakers, Steven Laureys, Haibo Di

**Affiliations:** 1 International Vegetative State and Consciousness Science Institute, Hangzhou Normal University, Hangzhou, China; 2 Coma Science Group, Cyclotron Research Centre and Neurology Department, University and University Hospital of Liege, Liege, Belgium; 3 Rehabilitation Center for Brain Damage, Wujing Hospital of Hangzhou City, Hangzhou, China

**Keywords:** Localisation to sound, Auditory localisation, Vegetative state, Unresponsive wakefulness syndrome, Minimally conscious state, Own name, Disorders of consciousness

## Abstract

**Background:**

At present, there is no consensus on how to clinically assess localisation to sound in patients recovering from coma. We here studied auditory localisation using the patient’s own name as compared to a meaningless sound (i.e., ringing bell).

**Methods:**

Eighty-six post-comatose patients diagnosed with a vegetative state/unresponsive wakefulness syndrome or a minimally conscious state were prospectively included. Localisation of auditory stimulation (i.e., head or eyes orientation toward the sound) was assessed using the patient’s own name as compared to a ringing bell. Statistical analyses used binomial testing with bonferroni correction for multiple comparisons.

**Results:**

37 (43%) out of the 86 studied patients showed localisation to auditory stimulation. More patients (n=34, 40%) oriented the head or eyes to their own name as compared to sound (n=20, 23%; p<0.001).

**Conclusions:**

When assessing auditory function in disorders of consciousness, using the patient’s own name is here shown to be more suitable to elicit a response as compared to neutral sound.

## Background

At present, there is no consensus on what auditory stimulus should be employed for the assessment of localisation to sound in disorders of consciousness such as the “vegetative state” (now also coined unresponsive wakefulness syndrome; VS/UWS [1]) and the minimally conscious state (MCS) [2]. Indeed, several behavioural “coma scales” use different stimuli to evaluate auditory localisation. For instance, the Coma Recovery Scale-Revised (CRS-R), the Sensory Modality Assessment Rehabilitation Technique and the Western Neuro-Sensory Stimulation Profile leave the choice open between several auditory stimuli (e.g., noise, voice). The Coma/Near Coma Scale requests to use “5s of bell ringing”, and the Wessex Head Injury Matrix uses a noise (bell, whistle or buzzer) and “a person talking” (for a review, see [3]).

We here propose to use the patient’s own name (as compared to a meaningless noise) in the assessment of localisation to sound. The own name is intrinsically meaningful for each of us because of its personal significance, emotional content and repetition along life. Beyond our day-to-day experience, the extreme salience of being presented one's own name was highlighted in various experimental and clinical studies. Some of these suggest that self-referential stimuli are so potent that they can "capture attention and subsequently bring the stimulus into awareness" [4]. In everyday social interactions, auto-referential stimuli give rise to a sense of self-awareness, as reflected in the cocktail party phenomenon when hearing our own name [5]. This particularly easy detection in usual laboratory experiments with healthy participants is consistent with research that showed powerful detection of the own name in situations of reduced consciousness [6,7]. The aim of the present study is to determine whether the assessment of localisation to sound in patients recovering from coma is influenced by the choice of the auditory stimulus.

## Methods

Eighty-six patients recovering from coma were prospectively assessed free of sedative drugs. Each patient was studied in a sitting position and a standardized arousal facilitation protocol (i.e., deep pressure stimulations from the facial musculature to the toes) was employed if needed in order to prolong the length of time the patient maintained arousal [8]. Localisation to sound was evaluated using a standardized methodology as described in the CRS-R [8]. In brief, an auditory stimulus (bell and patient’s own name) was presented from the right and from the left side while the examiner stood next to the patient but out of view. Stimuli were matched for intensity and duration of presentation, and were presented twice for each side. The order of presentation was randomized using “random number” procedure in Excel. Localisation to auditory stimulation was defined as head or eyes orientation toward the location of the stimulus on both trials for at least one side. Special care was made not to present stimuli when spontaneous eye or head movements were occurring. Clinical diagnosis was made according to the Aspen workgroup criteria for disorders of consciousness [2] and based on the CRS-R assessments [8] performed by two trained and experienced neuropsychologists. Note that according to these guidelines auditory localisation is compatible with the diagnosis of VS/UWS. The study was approved by the Ethics Committee of Hangzhou Normal University and Wujing Hospital which complies with the Code of Ethics of the World Medical Association (Declaration of Helsinki). Informed consents were obtained by the patient’s legal surrogates.

Differences between localisation as assessed by bell or patient's own name were measured using binomial testing (Stata Statistical Software; Release 11.2. College Station, TX: StataCorp LP 2009). Bonferroni correction was applied for multiple comparisons. Results were considered significant at p<0.01.

## Results

**Figure 1:**
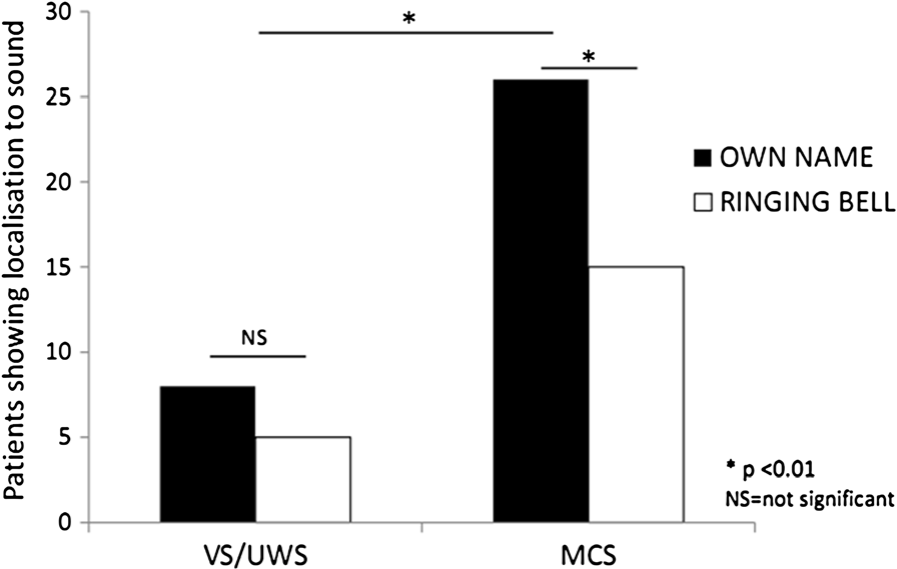
**Auditory localisation.** Number of patients in vegetative/unresponsive state (VS/UWS) and minimally conscious state (MCS) showing localisation to sound (n=37) as a function of the employed stimulus (own name in black and ringing bell in white).

Out of 86 patients (67 men; mean age 46 (SD 17) years), 47 (55%) were diagnosed in VS/UWS [1] and 37 (45%) were in MCS. Median time between injury and assessment was 5 months (IQR: 3 – 13 months). Aetiology was traumatic in 53 (61%) and non-traumatic in 33 (39%) patients. 37 (43%) out of the 86 studied patients showed localisation to auditory stimulation. Overall, more patients (n=34, 40%) oriented the head or eyes to the own name as compared to sound (n=20, 23%; p<0.001) (Table 1). MCS patients localized more often to their own name as compared to sound (p<0.001). This effect was not significant in the VS/UWS group (p>0.05) (Figure 1).

**Table 1:** Auditory localisation according to diagnosis and aetiology

**Diagnosis**	**Localisation response**				**Aetiology**		**Total**
	**Own name**	**Bell**	**Both**	**None**	**Traumatic**	**Non traumatic**	
VS/UWS	4 (5%)	1 (1%)	4 (5%)	38 (44%)	26 (30%)	21 (25%)	47
MCS	13 (15%)	2 (2%)	13 (15%)	11 (13%)	27 (31%)	12 (14%)	39
Total	17 (20%)	3 (3%)	17 (20%)	49 (57%)	53 (61%)	33 (39%)	86

Number of patients showing localisation to the own name, ringing bell or both as a function of diagnosis [vegetative state (VS/UWS) versus minimally conscious state (MCS)] and aetiology.

Tables 2 and 3 show the clinical data for each patient (MCS and VS/UWS patient groups respectively). Localisation preference was not different depending on aetiology or time since insult (p>0.05). The overall behavioural responsiveness assessed by the CRS-R total score tended to be higher when patients localized both stimuli than when they did not show any localisation (Tables 2 and 3). For instance, MCS patients showing both responses to their own name and to the bell had a CRS-R total score between 9 and 18 whereas MCS patients showing no localisation had a score between 6 and 10. Patients localizing to their own name only (or bell only) showed intermediate CRS-R total scores. In the 37 patients showing localisation, 9 patients were considered as being in VS/UWS according to the CRS-R criteria (i.e., they showed no response to command, no orientation to pain and no visual tracking) - 4 of these patients showed orientation to the own name but not to sound.

**Table 2:** Clinical data of the MCS patients

**Patient**	**Gender**	**Aetiology**	Time since injury*	CRS-R score**	**Auditory localisation**
MCS1	male	trauma	7	6	none
MCS2	male	trauma	73	7	none
MCS3	male	trauma	21	7	none
MCS4	female	trauma	20	7	none
MCS5	male	trauma	155	8	none
MCS6	female	trauma	160	8	none
MCS7	female	trauma	21	8	none
MCS8	male	trauma	205	9	none
MCS9	male	trauma	20	10	none
MCS10	male	stroke	51	10	none
MCS11	male	trauma	45	10	none
MCS12	female	trauma	9	8	bell
MCS13	male	stroke	32	9	bell
MCS14	male	trauma	55	8	own name
MCS15	male	trauma	11	8	own name
MCS16	male	trauma	221	8	own name
MCS17	male	trauma	150	9	own name
MCS18	male	trauma	40	9	own name
MCS19	female	stroke	14	9	own name
MCS20	male	stroke	61	10	own name
MCS21	male	trauma	22	10	own name
MCS22	male	trauma	19	13	own name
MCS23	male	trauma	7	13	own name
MCS24	female	trauma	54	14	own name
MCS25	male	trauma	291	14	own name
MCS26	male	stroke	115	16	own name
MCS27	male	anoxia	50	9	both
MCS28	female	trauma	7	10	both
MCS29	male	trauma	13	10	both
MCS30	male	trauma	121	10	both
MCS31	male	trauma	33	11	both
MCS32	male	stroke	13	11	both
MCS33	male	trauma	12	12	both
MCS34	male	stroke	9	13	both
MCS35	female	stroke	22	15	both
MCS36	male	anoxia	135	16	both
MCS37	male	stroke	6	16	both
MCS38	male	anoxia	57	17	both
MCS39	male	trauma	3	18	both

*Time since injury in weeks, ** Total score of the Coma Recovery Scale-Revised (minimum 0, maximum 23).

**Table 3:** Clinical data of the VS/UWS patients

**Patient**	**Gender**	**Aetiology**	Time since injury*	CRS-R score**	**Auditory localisation**
VS/UWS1	male	stroke	9	2	none
VS/UWS2	male	anoxic	6	3	none
VS/UWS3	male	trauma	34	3	none
VS/UWS4	male	trauma	17	3	none
VS/UWS5	male	stroke	66	4	none
VS/UWS6	male	anoxic	82	4	none
VS/UWS7	male	trauma	39	4	none
VS/UWS8	male	trauma	13	4	none
VS/UWS9	female	anoxia	4	4	none
VS/UWS10	male	stroke	13	5	none
VS/UWS11	male	anoxia	89	5	none
VS/UWS12	male	stroke	5	5	none
VS/UWS13	female	trauma	8	5	none
VS/UWS14	male	trauma	189	5	none
VS/UWS15	female	stroke	5	5	none
VS/UWS16	male	stroke	3	5	none
VS/UWS17	female	trauma	68	6	none
VS/UWS18	male	trauma	76	6	none
VS/UWS19	male	trauma	36	6	none
VS/UWS20	male	trauma	13	6	none
VS/UWS21	male	trauma	22	6	none
VS/UWS22	male	trauma	8	6	none
VS/UWS23	male	stroke	21	6	none
VS/UWS24	male	trauma	9	6	none
VS/UWS25	male	stroke	16	6	none
VS/UWS26	male	trauma	34	6	none
VS/UWS27	male	trauma	19	6	none
VS/UWS28	male	trauma	12	6	none
VS/UWS29	female	stroke	11	6	none
VS/UWS30	male	trauma	70	6	none
VS/UWS31	male	anoxia	413	6	none
VS/UWS32	male	trauma	28	6	none
VS/UWS33	male	trauma	34	7	none
VS/UWS34	male	trauma	24	7	none
VS/UWS35	female	trauma	11	7	none
VS/UWS36	male	stroke	9	7	none
VS/UWS37	female	stroke	13	7	none
VS/UWS38	male	trauma	10	7	none
VS/UWS39	female	anoxic	16	4	bell
VS/UWS40	male	anoxic	557	8	bell
VS/UWS41	male	stroke	39	5	own name
VS/UWS42	female	trauma	18	6	own name
VS/UWS43	male	anoxia	23	7	own name
VS/UWS44	female	anoxia	15	7	own name
VS/UWS45	female	trauma	20	6	both
VS/UWS46	male	trauma	14	7	both
VS/UWS47	male	trauma	38	8	both

*Time since injury in weeks, **Total score of the Coma Recovery Scale-Revised (minimum 0, maximum 23).

## Discussion

Our data show that the assessment of localisation to sound depends on what stimulus is employed. MCS patients tend to best orient to their own name as compared to a meaningless loud sound (i.e., ringing bell). Indeed, one’s own name is a piece of information that we use to process in the auditory modality from infancy: 4–5 month-old infants are able to recognize the sound pattern of their own names [9]. In end-stage demented patients, it has also been shown that perception of the own name deteriorates well after perception of time, place and recognition [10]. Similarly, after general anaesthesia, the patient’s reactivity to the own name occurs first, before reactivity to pain and noise [11]. In MCS patients, clinical experience learns that behavioural responses to auto-referential stimuli such as the own face are amidst the first signs heralding further recovery of consciousness [12]. Event-related potential studies have also shown that hearing one’s own name, as compared to meaningless noise, leads to an increased mismatch negativity response in patients with disorders of consciousness [6]. In addition, functional MRI studies assessing brain activation to the own name have reported activation of “self”-related brain regions (i.e., anterior cingulate and mesiofrontal cortices) depending of the level of consciousness in patients recovering from coma [7,13].

28% of the studied MCS patients (11/39) failed to show auditory localisation. Neurological assessment showed that 2 of these 11 patients (18%) had absent auditory startle, while 9 (82%) showed auditory-independent signs of consciousness. In line with previous studies, auditory impairment probably explains this finding [3].

Auditory localisation seems to be related to the patient’s overall behavioural responsivity: the more the patients are conscious, the more they tend to respond to both auditory stimuli. Moreover, our results showed that most of the patients who responded to the bell also responded to their own name (condition “both” in Table 1). Three patients however showed localisation to the bell but not to their own name. Even if they retained basic auditory processing, these three patients might not have been able to process language, and hence recognize their own name. Another explanation could be the presentation of the patient's own name as last stimulus, and hence fatigue might explain orientation to a bell in the absence of orientation to the own name.

One should note that the duration and the degree of the movement towards auditory stimulation were not taken into account to assess auditory localisation (as described in the CRS-R). This should nevertheless be investigated in future studies to allow differentiating between a brief movement and a sustained fixation following auditory stimulation. Indeed, the latter may potentially be considered as a sign of consciousness, as it is the case for visual and tactile localisation (e.g., visual pursuit and localisation to noxious stimulation items in the CRS-R). Such responses may also be worth exploring further using neuroimaging techniques such as fMRI and EEG in order to compare the behavioral responses and the underlying cerebral networks involved when hearing the person's name being called.

## Conclusions

Our findings emphasize the clinical importance of using the patient's own name when performing bedside testing of localisation to sound, adding to previous studies the importance of using auto-referential stimuli in patients with disorders of consciousness (i.e., the use of a mirror in the assessment of visual tracking [12]).

## Abbreviations

VS/UWS: Vegetative state/unresponsive wakefulness syndrome; MCS: Minimally conscious state; CRS-R: Coma recovery scale-revised; fMRI: Functional magnetic resonance imaging; EEG: Electroencephalography.

## Competing interests

The authors declare that they have no competing interests.

## Pre-publication history

The pre-publication history for this paper can be accessed here:

http://www.biomedcentral.com/1471-2377/13/27/prepub
